# Genome-wide identification and expression analysis of *CASPL* gene family in *Zea mays* (L.)

**DOI:** 10.3389/fpls.2024.1477383

**Published:** 2024-10-28

**Authors:** Baoping Xue, Zicong Liang, Dongyang Li, Yue Liu, Chang Liu

**Affiliations:** ^1^ College of Agronomy, Shenyang Agriculture University, Shenyang, China; ^2^ Department of Plant Sciences, College of Life Sciences, Wuhan University, Wuhan, China; ^3^ Institute of Applied Ecology, Chinese Academy of Sciences, Shenyang, China

**Keywords:** *Zea mays*, CASPL gene family, systematic evolution, expression pattern, biotic and abiotic stress

## Abstract

Casparian strip membrane domain proteins like (CASPL), exhibit profound associations with root development, stress responsiveness and mineral element uptake in plants. Nonetheless, a comprehensive bioinformatics analysis of the *ZmCASPL* gene family in maize remains unreported. In the study, we have identified 47 *ZmCASPL* members at the whole-genome level, systematically classifying them into six distinct groups. Furthermore, our analysis revealed that the same group of *ZmCASPL* contains similar gene structures and conserved motifs. Duplication events showed whole genome duplication (WGD) and tandem duplication (TD) contribute to the generation of the *ZmCASPL* gene family together in maize, but the former plays a more prominent role. Furthermore, we observed that most *ZmCASPL* genes contain MYB-binding sites (CAACCA), which are associated with the Casparian strip. Utilizing RNA-seq data, we found that *ZmCASPL21* and *ZmCASPL47* are specifically highly expressed only in the roots. This finding implies that *ZmCASPL21* and *ZmCASPL47* may be involved in the Casparian strip development. Additionally, RNA-seq analysis illuminated that drought, salt, heat, cold stresses, low nitrogen and phosphorus conditions, as well as pathogen infection, significantly impact the expression patterns of *ZmCASPL* genes. RT-qPCR revealed that *ZmCASPL 5/13/25/44* genes showed different expression patterns under PEG and NaCl treatments. Collectively, these findings provide a robust theoretical foundation for further investigations into the functional roles of the *ZmCASPL* gene family in maize.

## Introduction

As global climate change intensifies, extreme weather events have become increasingly ubiquitous, with drought and soil salinization emerging as prominent constraints that significantly limit crop growth and yield. Maize, a globally pivotal food crop, is directly impacted by these abiotic stresses, resulting in diminished yields and compromised quality (Muthuvel et al., 2023). Consequently, a profound investigation into the physiological mechanisms, genetic attributes, and adaptive responses of maize under drought and salt stress is of paramount significance for the development of novel drought-tolerant and -drought-tolerant maize varieties. It is worth noting that plants have evolved elaborate defense strategies to cope with these stresses, such as oxidative burst, the deposition of lignin and callose within cell walls, and adaptive root development (Kunkel and Brooks, 2002; Jones and Dangl, 2006; Zhang et al., 2007; Singh et al., 2015; Zheng et al., 2024). Roots, as the primary organs for water and mineral uptake in plants, exhibit remarkable adaptability and plasticity under stress conditions (Hu et al., 2022; Zheng et al., 2024; Li et al., 2024). Specifically, under drought conditions, plants augment their water acquisition capabilities by promoting root growth and development, thereby enhancing plants drought tolerance (Chen et al., 2022; Wang et al., 2024). Similarly, roots play a pivotal role in conferring tolerance to salt stress (Yu et al., 2022a).

The main component of the Casparian strip (CS) is lignin, deposited between endodermal cells (Naseer et al., 2012). This CS serves as an apoplastic transport barrier, effectively preventing the mineral nutrients free into and out of the root stele (Barberon et al., 2016; Barberon, 2017). Therefore, it plays a pivotal function in the selective uptake of nutrients and the maintenance of ion homeostasis within plants. Previous studies have confirmed that under salt stress conditions, the CS effectively blocks the influx of excessive Na^+^ ions from the soil into the stele (Karahara et al., 2004). Furthermore, in maize roots, the CS forms earlier thickens and widens under salt stress (Karahara et al., 2004). Similar phenomena have also been observed in other crops such as rice and cotton (Krishnamurthy et al., 2009), suggesting that this adaptive mechanism is widespread among plants facing salt stress.

Recently, the molecular mechanisms of Casparian strip formation have been studied in *Arabidopsis*. CASPs (Casparian Strip Membrane Domain Proteins) are first identified family of membrane proteins that are associated with the CS (Roppolo et al., 2011). These transmembrane proteins, which are hallmarked by their possession of four transmembrane domains, exhibit a unique expression pattern, specifically localized to the CS within the root endodermis (Roppolo et al., 2011). In *Arabidopsis*, the CASP family comprises five members, designated CASP1-CASP5, which redundantly regulate Casparian strip formation (Roppolo et al., 2011). When knockout of *atcasp1* or *atcasp3* alone did not affect CS formation, and only the *atcasp1 atcasp3* double mutant observed defects in CS formation (Roppolo et al., 2011). Compared to *Arabidopsis*, When knockout of OsCASP1 alone observed defects in CS formation in rice. CASPs proteins further recruit some enzymes and cofactors to synergistically regulate lignin polymerization, such as respiratory burst oxidase homolog F (RBOHF), the enhanced suberin 1 (ESB1), Peroxidase 64 (PER64) and UCLACYANIN 1 (UCC1) (Roppolo et al., 2011; Hosmani et al., 2013; Kalmbach et al., 2017; Reyt et al., 2021). These enzymes and co-factors are positively regulated by the transcription factor *MYB36* (Kamiya et al., 2015; Liberman et al., 2015). Additionally, the CIF1/2 (small peptide) -SGN3 (leucine-rich repeat receptor-like kinase SGN3)-SGN1 (receptor-like cytoplasmic kinase (RLCK)) signaling pathway is considered to monitor the integrity of the Casparian strip (Pfister et al., 2014; Okuda et al., 2020).

CASPLs (CASP-like) proteins are ubiquitous in plants, and are characterized by a protein architecture comprising four transmembrane domains, two intracellular loops, one extracellular loop, as well as N-terminal and C-terminal residues, exhibiting a high degree of homology with the CASPs family proteins (Su et al., 2023). Furthermore, the transmembrane domains of CASPLs family proteins share considerable similarity with proteins carrying the MARVEL domain, thus belonging to the MARVEL family of proteins. Notably, the protein sequences of the first transmembrane domain (TM1) and the third transmembrane domain (TM3) are highly conserved (Sánchez et al., 2002). With the release of plant genome sequences, the presence of *CASPL* genes has been discovered in plants such as *Arabidopsis* (Roppolo et al., 2011), rice, *Gossypium arboretum* (Wang et al., 2020), and *Pogostemon cablin* (Su et al., 2023). Currently, there is limited research on the functional aspects of the CASPLs family genes. In *Gossypium arboretum*, *GaMYB36* was found to regulate lateral root growth by targeting *GaCASP27* (Wang et al., 2020). Most *CASPL* genes can respond to p-hydroxybenzoic acid (p-HBA) stress treatment, such as 27 *PatCASPL* genes and 30 *PatCASPL* were upregulated and down-regulated, respectively (Su et al., 2023). Yang et al. identified a *ClCASPL* gene (*ClCASPL41*) induced by cold stress in watermelon. Interestingly, the orthologous gene in *Arabidopsis* (*AtCASPL4C1*) was also found to play a key role in cold tolerance (Yang et al., 2015). The knockout of *AtCASPL4C1* showed enhanced cold stress tolerance, while overexpressing *CICASPL* resulted in increased sensitivity to cold stress in *Arabidopsis* (Yang et al., 2015). The formation of the cuticular wax structure in maize necessitates *ZmSRL5*, which, by preserving the integrity of this wax layer, enhances the plant’s ability to withstand drought conditions (Pan et al., 2020).

Comprehending the roles of *CASPL* family genes in biological and physiological processes offers a viable avenue for analyzing and enhancing crop defenses against both biotic and abiotic stresses. However, there is a lack of systematic bioinformatics research on *CASPL* in maize. Consequently, this study aims to delve into the potential functions of maize *CASPL* family genes through genome-wide identification and gene expression analysis. Here, we identified 47 *CASPL* genes from the maize genome. Subsequently, we performed a systematic analysis encompassing multiple sequence alignments, gene structures, conserved domains, chromosomal locations, gene duplications and phylogenetic relationships, aiming to unravel the evolutionary history of maize CASPL protein expansion. Additionally, we evaluated the expression patterns of *CASPLs* genes in different tissues and their responses to various abiotic stresses. This study serves as a foundation for understanding the roles of *CASPL* genes in maize response to abiotic stresses like drought and salt and paves new avenues for future research on the *CASPL* gene family.

## Results

### Identification of ZmCASPL family members

**Figure 1:**
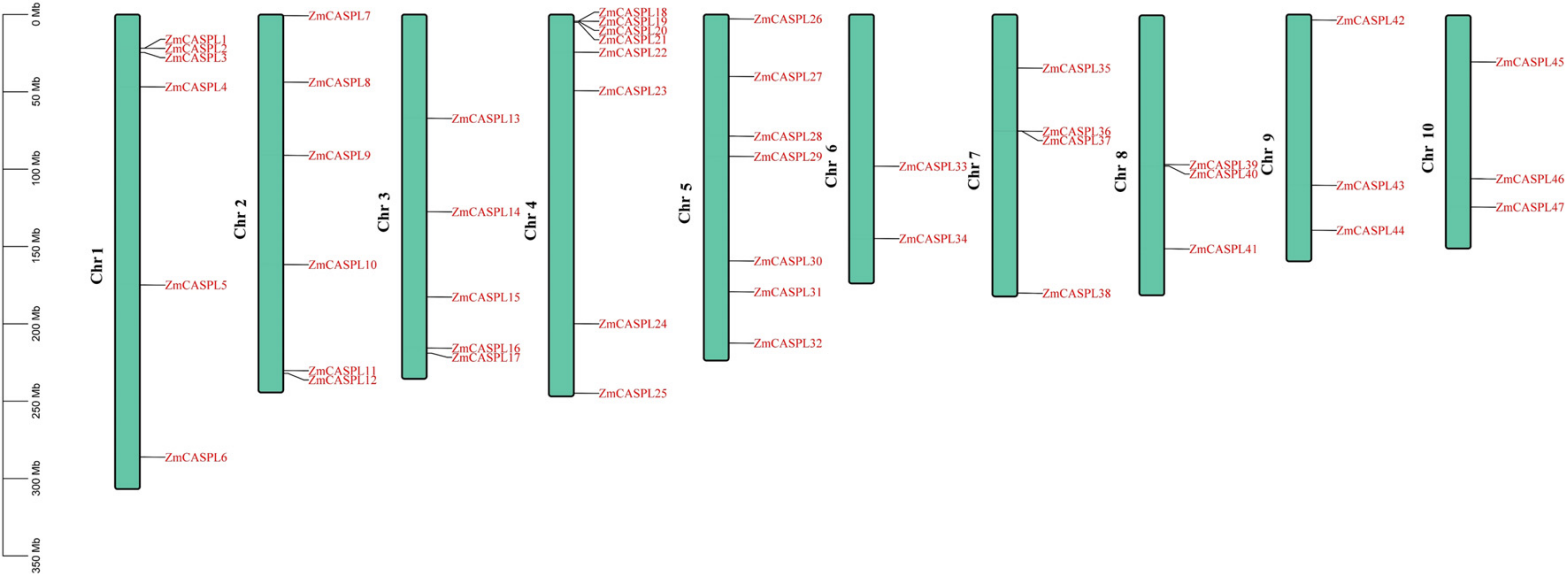
The chromosome location of *ZmCASPL* genes at *Z. mays*. The chromosomal distribution information of the *ZmCASPL* genes was generated from the GFF file information of *Z. mays*. This image was visualized by Tbtools software.

In this study, a total of 47 *ZmCASPL* members were found by hidden Markov model (HMM) (E-value<1e^-5^) and BLASTP methods (E-value<1e^-5^) (**Supplementary Table 1**). These genes are systematically named *ZmCASPL1*-*ZmCASPL47*, based on their location on the chromosome (**Figure 1**). Notably, the distribution of 47 *ZmCASPL* genes across the 10 chromosomes (Chr) of maize was found to be uneven (**Figure 1**). Specifically, Chr4, Chr5, Chr1 and Chr2 contain the highest number of *ZmCASPL* genes, with counts 8, 7, 6 and 6, respectively. On Chr4, an intriguing pattern emerged, with *ZmCASPL18*, *ZmCASPL19*, *ZmCASPL20* and *ZmCASPL21* genes occurring in clusters. This is followed by Chr 3, which contains five *ZmCASPL* genes. Interestingly, Chr 7, 8, 9, and 10 each contained an equal number of four *ZmCASPL* genes, while Chr 6 contains the least with two only genes.

Furthermore, a detailed analysis of the physicochemical properties of ZmCASPL protein was undertaken. As shown in **Supplementary Table 1**, the amino acid counts of ZmCASPL varied substantially, ranging from 105 (ZmCASPL37) to 390 (ZmCASPL17), with an average of 206.72. Notably, only 30% (16 CASPL) of CASPL proteins exhibited amino acid counts greater than this average. The molecular weight of ZmCASPL spanned a wide range, from 11349.43 Da (ZmCASPL37) to 40609.75 Da (ZmCASPL17). Additionally, the pI of ZmCASPL proteins ranged from 4.61 to 10.01, with an average of 8.24, and most of them were greater than 7, indicating that ZmCASPL proteins are basic proteins.

Interestingly, our analysis revealed that the instability index of 19 ZmCASPL proteins surpassed 40, while the remainder fell below this threshold, suggesting that the majority CASPL proteins may be stable proteins in maize. The grand average of hydropathicity of ZmCASPL16, ZmCASPL17, ZmCASPL25 and ZmCASPL28 were less than zero, on the contrary, 43 ZmCASPL are greater than zero (**Supplementary Table 1**), implying most ZmCASPL are hydrophobic proteins. These findings contribute valuable insights into the structural and functional characteristics of the ZmCASPL protein.

### Multiple sequence alignment and evolutionary analysis

**Figure 2:**
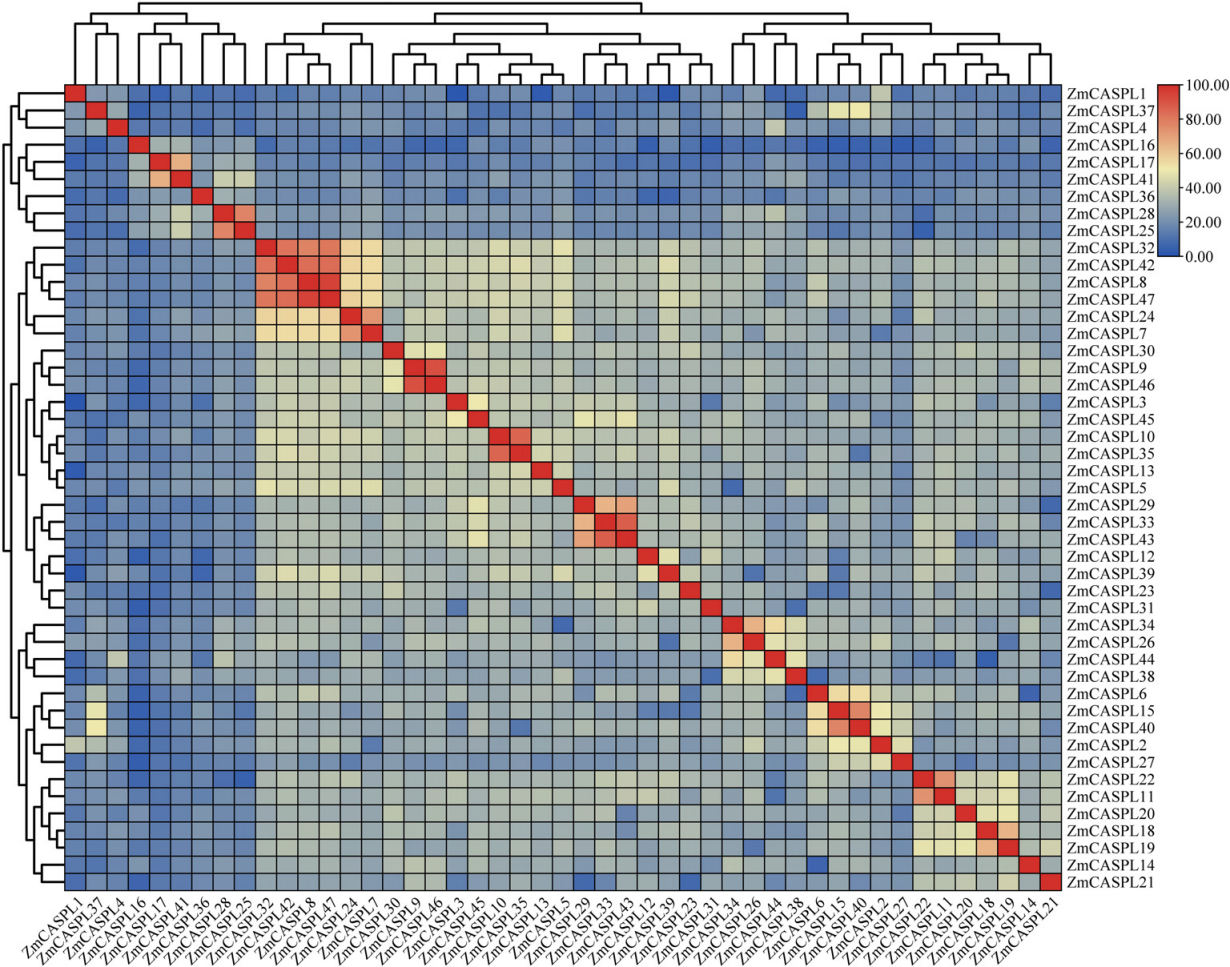
Alignment of multiple protein sequences of ZmCASPL. Red and blue boxes indicate high and low similarity of ZmCASPL proteins, respectively.

To delve into the evolutionary mechanism of the ZmCASPL proteins, we first performed a multiple sequence alignment of ZmCASPL proteins. Subsequently, we constructed evolutionary trees encompassing 39 AtCASPL and 47 ZmCASPL proteins, aiming to elucidate their phylogenetic relationships. As shown in **Figure 2**, a notable degree of homology is observed among ZmCASPL8, ZmCASPL32, ZmCASPL42, and ZmCASPL47, with amino acid similarity indices spanning from 71.68% to 83.85%. Specifically, the amino acid similarity between ZmCASPL42 and ZmCASPL47 stands out at 91.59%, while that between ZmCASPL42 and ZmCASPL32 reaches 85.78%, and between ZmCASPL33 and ZmCASPL43, it is 87.63% (**Figure 2**). Moreover, the amino acid similarity of ZmCASPL42 and ZmCASPL47 was 91.59%, ZmCASPL42 and ZmCASPL47 was 85.78%, and ZmCASPL33 and ZmCASPL43 was 87.63%. These findings imply a potential functional redundancy among these *ZmCASPL* genes, suggesting they may share overlapping roles in biological processes.

**Figure 3:**
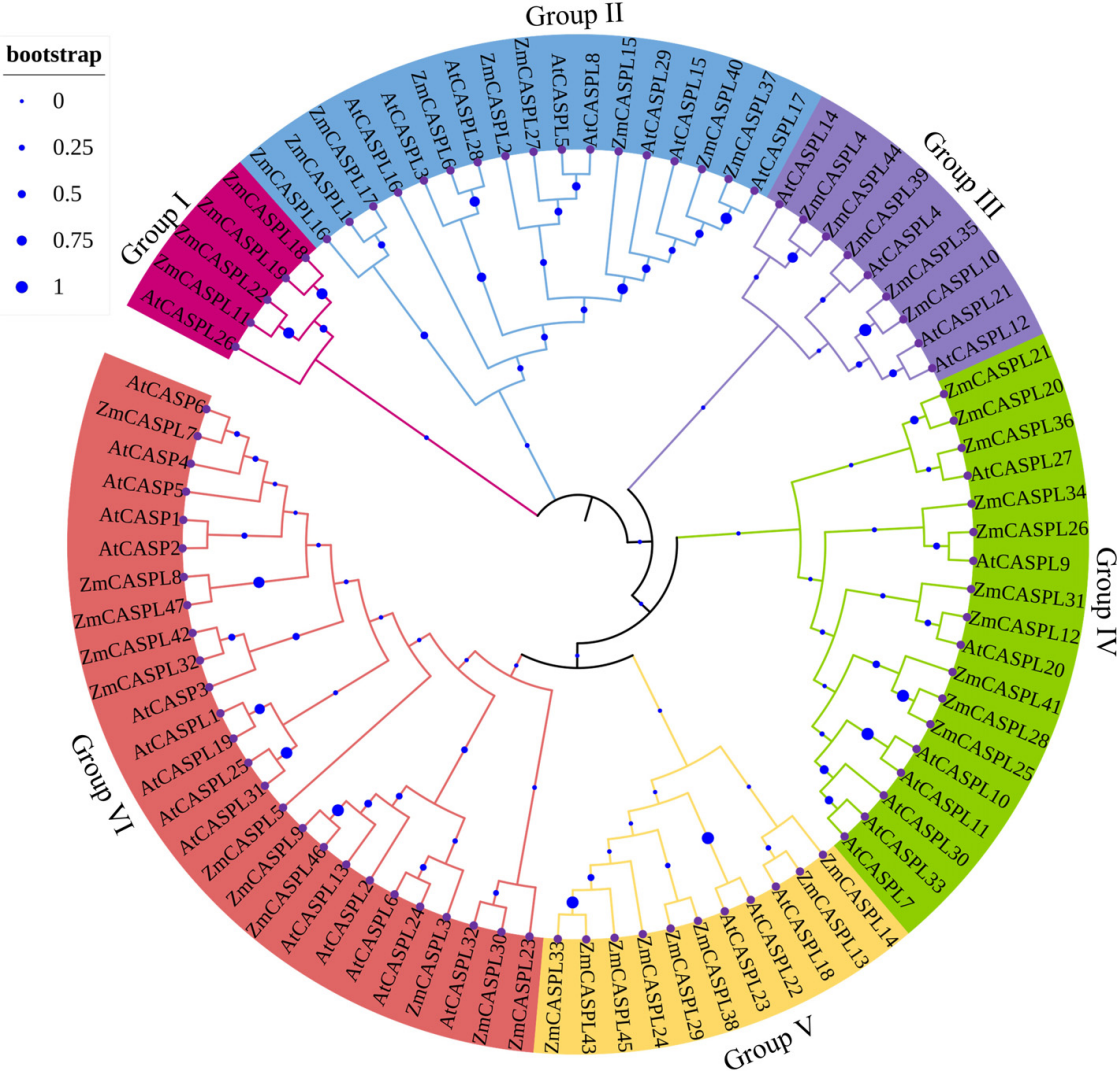
The phylogenetic trees were generated of 47 ZmCASPL and 39 AtCASPL proteins by MEGA 7 (method: Neighbor-Joining; parameter: bootstrap values of 1000 replicates). Magenta, blue, purple, green, orange, and red represents Group I, Group II, Group III, Group IV, Group V and Group VI, respectively.

The phylogenetic tree revealed a clear classification of the 39 AtCASPL and 47 ZmCASPL proteins into six distinct subfamilies: Group I, Group II, Group III, Group IV, Group V and Group VI (**Figure 3**). Notably, the Group VI subfamily is the largest, comprising 10 ZmCASPL and 15 AtCASPL, indicative of its significant evolutionary and functional significance. Following closely behind are Group II and Group IV, each containing 9 ZmCASPL and 8 AtCASPL proteins, and 10 ZmCASPL and 8 AtCASPL proteins, respectively. In contrast, Group I contain a notably smaller number of proteins, including solely 4 ZmCASPL and 1 AtCASPL. Previous studies have shown that AtCASP1 and AtCASP3 and OsCASP1 can regulate endodermis CS formation in *Arabidopsis* and rice (Roppolo et al., 2011; Wang et al., 2019). As shown in **Figure 3**, we found that ZmCASPL32 and ZmCASPL42 were closely related to AtCASP1, implying that *ZmCASPL32* and *ZmCASPL42* genes might be involved in endodermal CS development and selective absorption of mineral elements in maize. Thus, our findings provide novel insights into the potential functional conservation and diversification within the *CASPL* gene family across plant species.

### Gene structure and conserved motif of ZmCASPL

**Figure 4:**
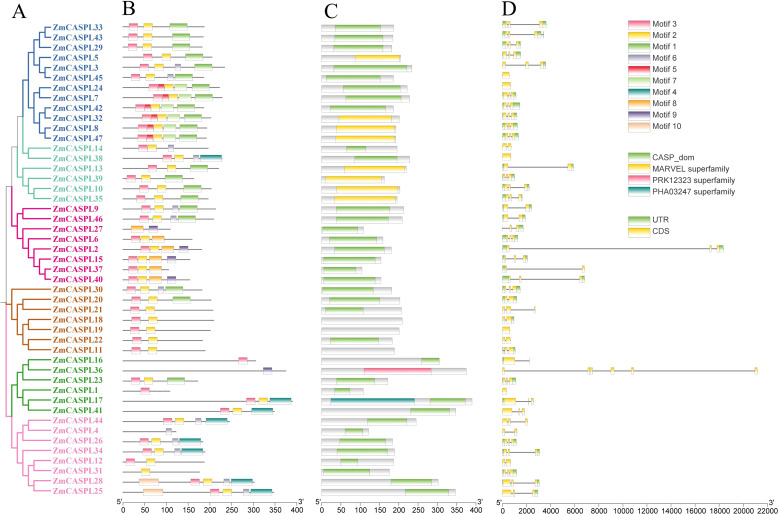
Phylogenetic tree, conserved motif, conserved domain and gene structure were analyzed of ZmCASPL. **(A)** Phylogenetic tree; **(B)** conserved motif; **(C)** conserved domain and **(D)** gene structure of ZmCASPL.

To further explore the evolutionary process of *ZmCASPL* gene family, the gene structure and conserved motifs of ZmCASPL were analyzed. As shown in **Figure 4**, a substantial majority (72%) of ZmCASPL proteins contain CASP domains, while ZmCASPL5, ZmCASPL8, ZmCASPL32, ZmCASPL47, ZmCASPL10, ZmCASPL13, ZmCASPL35, and ZmCASPL39 contain the MARVEL domains. Interestingly, we found that ZmCASPL17 consists of two domains in series, CASP and PHA00427. Furthermore, we also analyzed the gene structure of *ZmCASPL*, and most *ZmCASPL* genes contained three exons (57.45%), whereas Z*mCASPL1*, *ZmCASPL7*, *ZmCASPL16*, *ZmCASPL19*, *ZmCASPL24* and *ZmCASPL38* contained only one exon (**Figure 4**). It is worth noting that *ZmCASPL36* contained six exons (**Figure 4**). Additionally, *ZmCASPL4*/*9*/*12/14*/*18*/*20*/*22*/*46* contained two exons. Remarkably, we found that the same Group of ZmCASPL contains similar conserved motifs. Specifically, Motif 2 and Motif 3 were contained in 85% ZmCASPL members, whereas Motif 4 was unique to ZmCASPL17, ZmCASPL25/26/28/34/38/41/44 (**Figure 4**), which meant that they were quite conserved in the ZmCASPL proteins. ZmCASPL7/8/24/32/42/47 contain the most Motifs, consisting of Motif 1, Motif 2, Motif 3 Motif 5 and Motif 7, respectively. Collectively, these findings suggest that the ZmCASPL proteins in maize have undergone significant functional divergence during evolution, as evidenced by their diverse gene structures, exon-intron arrangements, and conserved motif compositions. This evolutionary plasticity likely contributes to the adaptability and versatility of maize in diverse environmental conditions.

### Duplication events analysis of *ZmCASPL*

**Figure 5:**
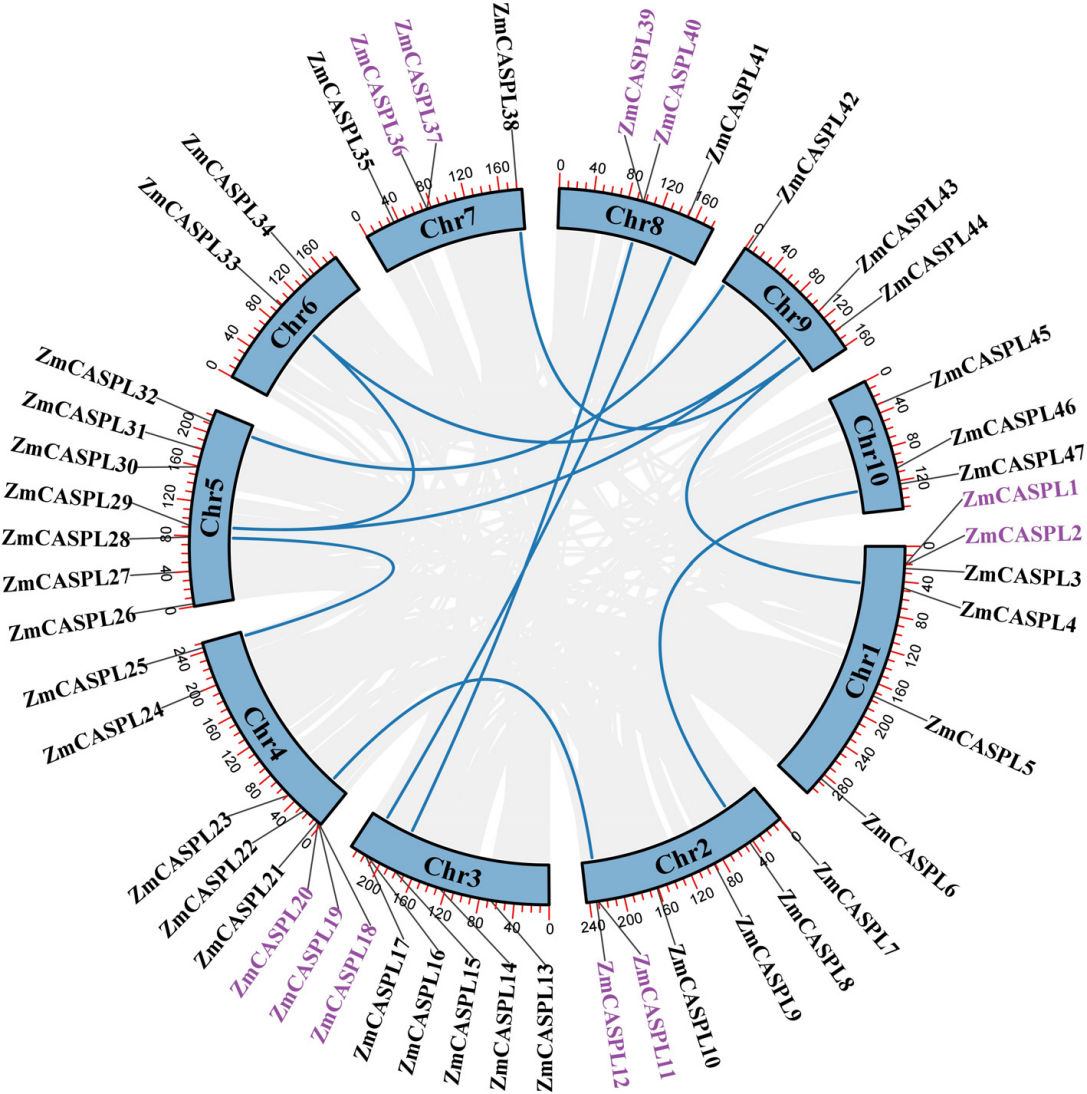
The synteny analysis of *ZmCASPL* genes in maize. The duplication events were analyzed of *ZmCASPL* genes in maize. Genes with purple color represented the TD and blue line represented the WGD genes.

The whole-genome duplication (WGD) and tandem duplication (TD) offer potential avenues for the production of new genes and functional evolution (Freeling, 2009; Panchy et al., 2016). Through WGD, organisms can acquire additional gene copies, subsequently enhancing opportunities for increased gene expression levels and functional expansion (Freeling, 2009). Meanwhile, TD creates multiple genes with similar functions on chromosomes, potentially facilitating the emergence of novel genes and driving the evolution of functionalities (Holland, 2013). To explore the mechanism of *ZmCASPL* gene family expansion in maize, we analyzed the duplication events of *ZmCASPL* genes. As shown in **Figure 5**, We identified 11 TD genes and 18 WGD genes in maize. These studies showed that TD and WGD contribute to the generation of *CASPL* gene family together in rice and *Arabidopsis*, with WGD playing a more prominent role. It is worth noting that *ZmCASPL29* has two homologous genes, *ZmCASPL33* and *ZmCASPL43*. These findings showed that TD and WGD contribute to the generation of *ZmCASPL* gene family together in maize, but the latter plays a more prominent role. Moreover, we also analyzed the Ka (nonsynonymous)/Ks (synonymous) ratios by Tbtools (Chen et al., 2023). As shown in **Supplementary Table 2**, the Ks/Ka ratio of most homologous gene pairs of *ZmCASPL* was less than 1, except *ZmCASPL11/ZmCASPL12*, suggesting that *ZmCASPL* gene under purifying selection.

### Cis-element analysis of *ZmCASPL* genes

**Figure 6:**
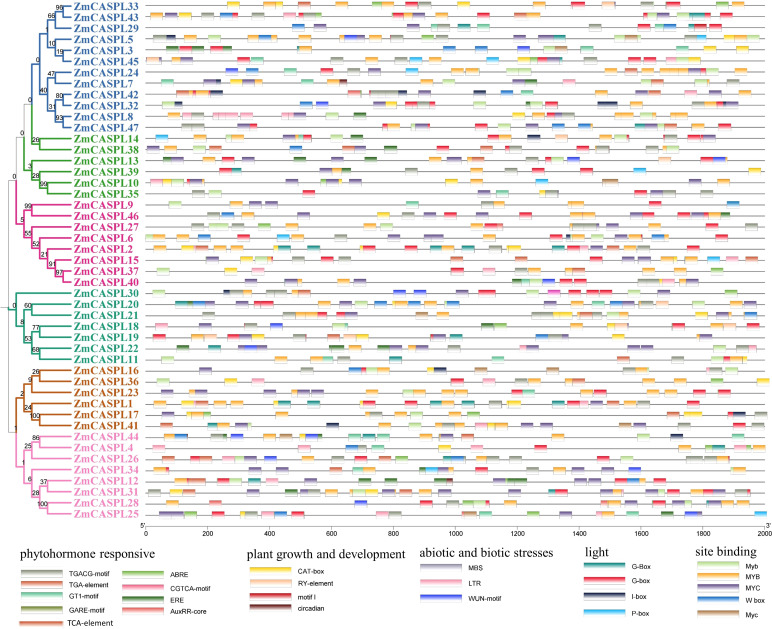
The cis-elements were predicted in 2000 bp promoter sequences of *ZmCASPL* genes. The Cis-elements was analyzed of *ZmCASPL* genes by Plantcare.

Promoters play a pivotal role in gene expression and functional regulation. To delve deeper into the potential regulatory functions of the *ZmCASPL* genes, we utilized the Plantcare online website to analyze the cis-elements within the 2k promoter region of the *ZmCASPL* gene. We identified a total of 26 cis-acting elements in the promoter regions of *ZmCASPL* genes, primarily encompassing hormone-responsive elements, growth and development-related elements, biotic and abiotic stress-related elements, site-binding-related elements, and light-responsive elements (**Figure 6**; **Supplementary Table 3**). Notably, 44 *ZmCASPL* genes were found to harbor ABA-responsive elements, suggesting a pivotal role for *ZmCASPL* genes in the ABA signaling pathway. Furthermore, *ZmCASPL11*/*17*/*18*/*19*/*20*/*21*/*32* uniquely possess both ABA-responsive and drought-responsive elements, indicating their potential to mediate drought stress responses via the ABA signaling pathway. Notably, *ZmCASPL18*, *ZmCASPL19*, and *ZmCASPL20* form a tandemly duplicated gene pair, suggesting that these genes may function cooperatively. Additionally, *ZmCASPL11*, *ZmCASPL24*, *ZmCASPL38*, *ZmCASPL43*, and *ZmCASPL45* not only contain jasmonic acid (JA)-responsive elements but also salicylic acid (SA)-responsive elements, hinting at their antagonistic roles in disease resistance in maize. Furthermore, we observed that most *ZmCASPL* genes contain MYB-binding sites (CAACCA), which are associated with the Casparian strip, further underscoring their potential functional implications. In summary, our findings provide valuable insights into the complex regulatory landscape of *ZmCASPL* genes, revealing their multifaceted roles in hormonal signaling, stress responses, and potentially, disease resistance, thereby contributing to the overall growth and development of maize.

### Tissue expression patterns *ZmCASPL* genes by RNA-seq

**Figure 7:**
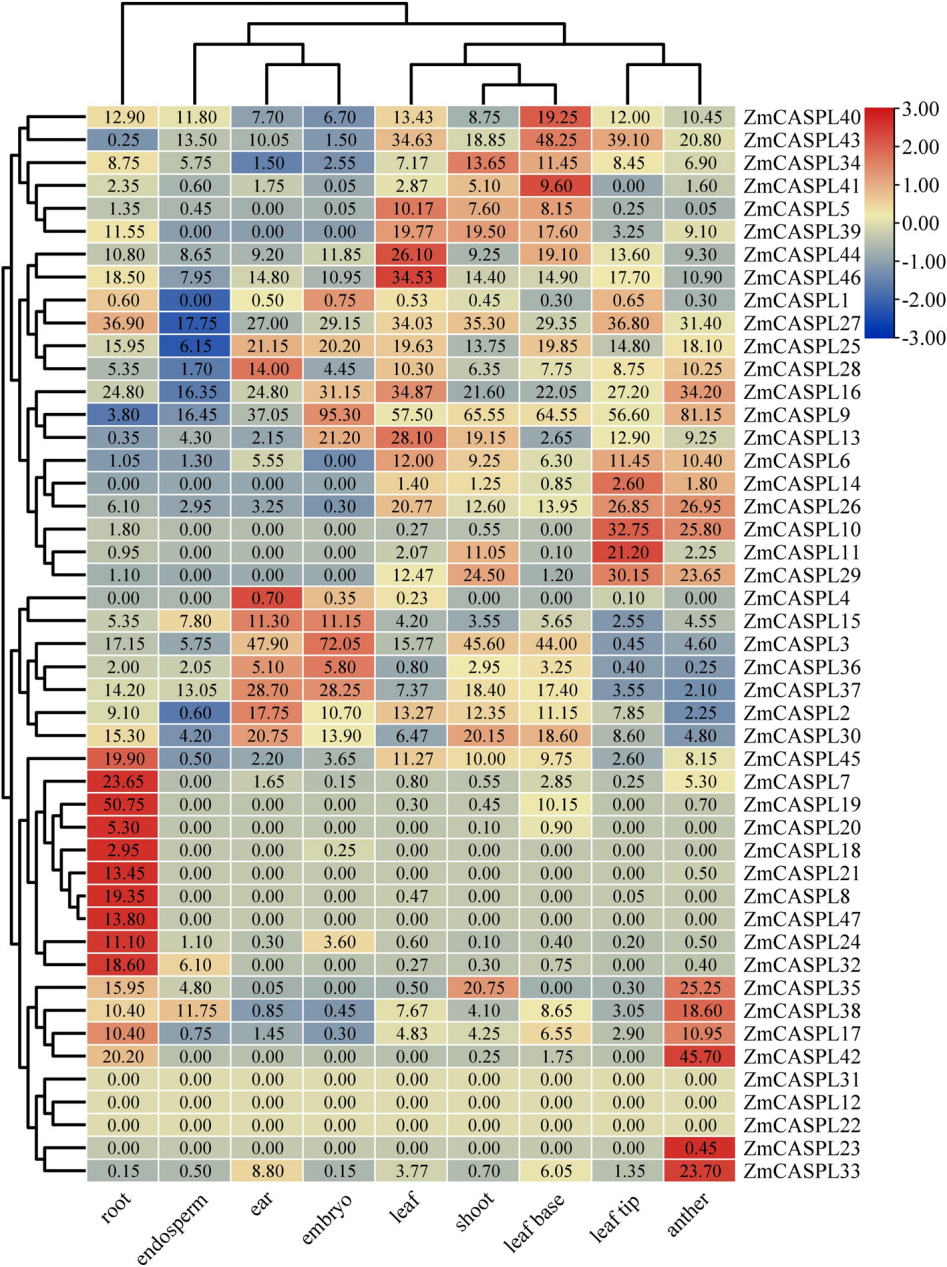
Expression pattern analysis of *ZmCASPL* genes in different tissues of maize. The expression pattern of *ZmCASPL* genes in different tissues (root, endosperm, ear, embryo, leaf, shoot, leaf base, leaf tip and anther). The heatmap is constructed by Tbtools software. Red and blue boxes indicate high and low expression levels of *ZmCASPL* genes, respectively.

The analysis of gene expression patterns provides profound insights into the regulatory mechanisms and biological functions of genes within organisms. To explore the expression patterns of *ZmCASPL* genes, we mined the RNA-seq data from nine organs (root, shoot, leaf base, leaf, leaf tip, ear, anther, endosperm and embryo) in maize (Yu et al., 2022b) (**Supplementary Table 4**). We found that *ZmCASPL* genes were widely expressed in various tissues of maize. As shown in **Figure 7**, among the 47 *ZmCASPL* genes, 43 were expressed in the roots, and 14 were highly expressed in the roots. It is worth mentioning that *ZmCASPL7*/*8*/*18*/*19*/*21*/*24*/*32*/*47* are specifically highly expressed in the root, and *ZmCASPL21* and *ZmCASPL47* is only highly expressed in the root. Interestingly, we found that the promoter region of *ZmCASPL21* and *ZmCASPL47* contains the “CAACCA” motif that is specifically bound by the transcription factor MYB36, suggesting that *ZmCASPL21* and *ZmCASPL47* may be involved in the Casparian strip development in maize. Notably, only some genes were highly expressed in other tissues. Such as, *ZmCASPL24*, *ZmCASPL32* and *ZmCASPL47* were highly expressed in anthers, while *ZmCASPL2*/*3/4*/*15*/*30/36/47* exhibited high expression in ears, and *ZmCASPL36* and *ZmCASPL47* in the leaf tip. Conversely, *ZmCASPL8*/*10*/*11/14*/*18/19*/*L20*/*21*/*24*/*29*/*32*/*39*/*47* exhibited rather no or low expression levels in all ear, endosperm and embryo. In addition, the *ZmCASPL12*, *ZmCASPL22* and *ZmCASPL31* genes were not detected in the tissues analyzed, and there are two possible reasons for this. One is that these genes may have specific expression patterns, and the other is that these genes may be non-functional pseudogenes. These results suggest that *ZmCASPL* genes may be involved in various stages of maize growth and development.

### The expression patterns of *ZmCASPL* genes in the different abiotic stress by transcriptome data

**Figure 8:**
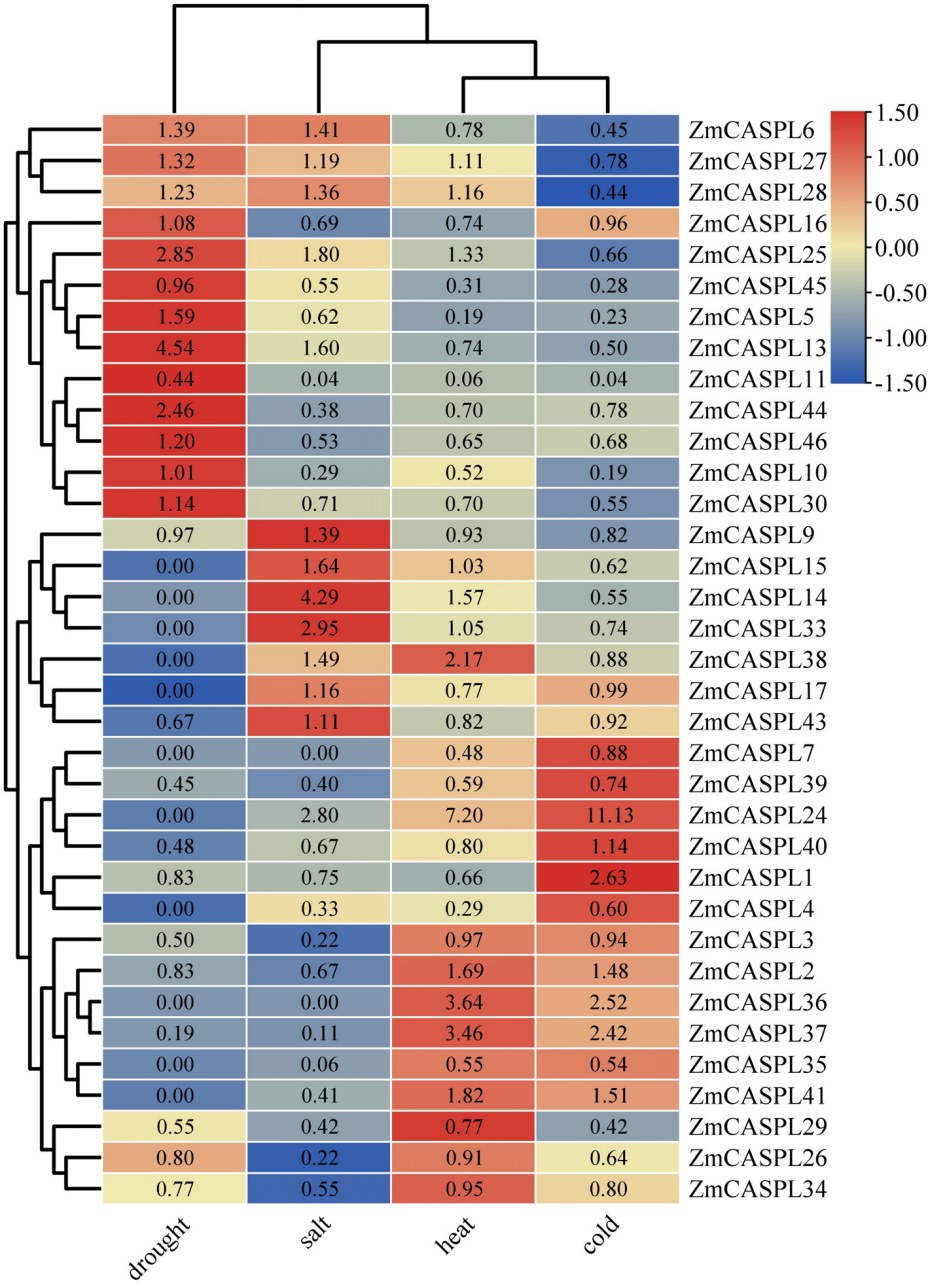
The expression pattern of *ZmCASPL* genes under drought, salt, heat and cold stresses by RNA-seq. The red and blue boxes indicate high and low expression levels of *ZmCASPL* genes, respectively.

Maize is one of the most important food crops in the world, among them, drought, extreme changes in environment temperature and land salinization and other abiotic stresses have become the key factors restricting maize yield and quality (Sun et al., 2023). It is worth noting that *CASPL* gene plays an important role in the process of salt and drought stress tolerance (Yang et al., 2015). To identify potential *ZmCASPL* genes that respond to these abiotic stresses, we analyzed transcriptome data of maize subjected to drought, heat, cold, and salt stresses (Yu et al., 2022b) (**Supplementary Table 5**). As shown in **Figure 8**, our analysis revealed that under drought stress, the expression levels of *ZmCASPL5*/*6/10/13/16/25/27/30* genes were significantly up-regulated, while the expression levels of *ZmCASPL3*/*29*/*34*/*3*7/*39*/*40/43* genes were down-regulated under drought stress. Notably, *ZmCASPL13*, *ZmCASPL25*, and *ZmCASPL44* exhibited particularly up-regulation (exceeding 2.46- to 4.54-fold). After salt, the expression levels of *ZmCASPL6/*9/*13/14/15/24*/*25*/*2*8/*33*/*38*/*43* were significantly up-regulated, whereas *ZmCASPL3*/*4*/*5*/*10*/*11*/*16*/*35*/*39*/*41*/*44*/*4546* showed marked down-regulated. Moreover, some *ZmCASPL* genes displayed opposing expression patterns under salt and drought stresses. For example, *ZmCASPL5*, *ZmCASPL30*, *ZmCASPL44*, and *ZmCASPL46* were down-regulated under salt stress but up-regulated under drought stress, whereas *ZmCASPL9* and *ZmCASPL43* exhibited the reverse trend. Interestingly, partial *ZmCASPLs* genes showed a similar trend after being subjected to heat and cold stress. For instance, the expression levels of *ZmCASPL2*, *ZmCASPL2*, *ZmCASPL24*, *ZmCASPL36*, *ZmCASPL37* and *ZmCASPL41* were down-regulated under drought stress, while the expression levels of *ZmCASPL4*/*5/10/11/35*/*45* genes were up-regulated under heat and cold stress, respectively. This comprehensive analysis underscores the complexity and diversity of *ZmCASPL* gene expression in response to various abiotic stresses, highlighting their potential as targets for improving maize stress tolerance.

### The expression patterns of *ZmCASPL* genes in low nitrogen and phosphorus stresses

**Figure 9:**
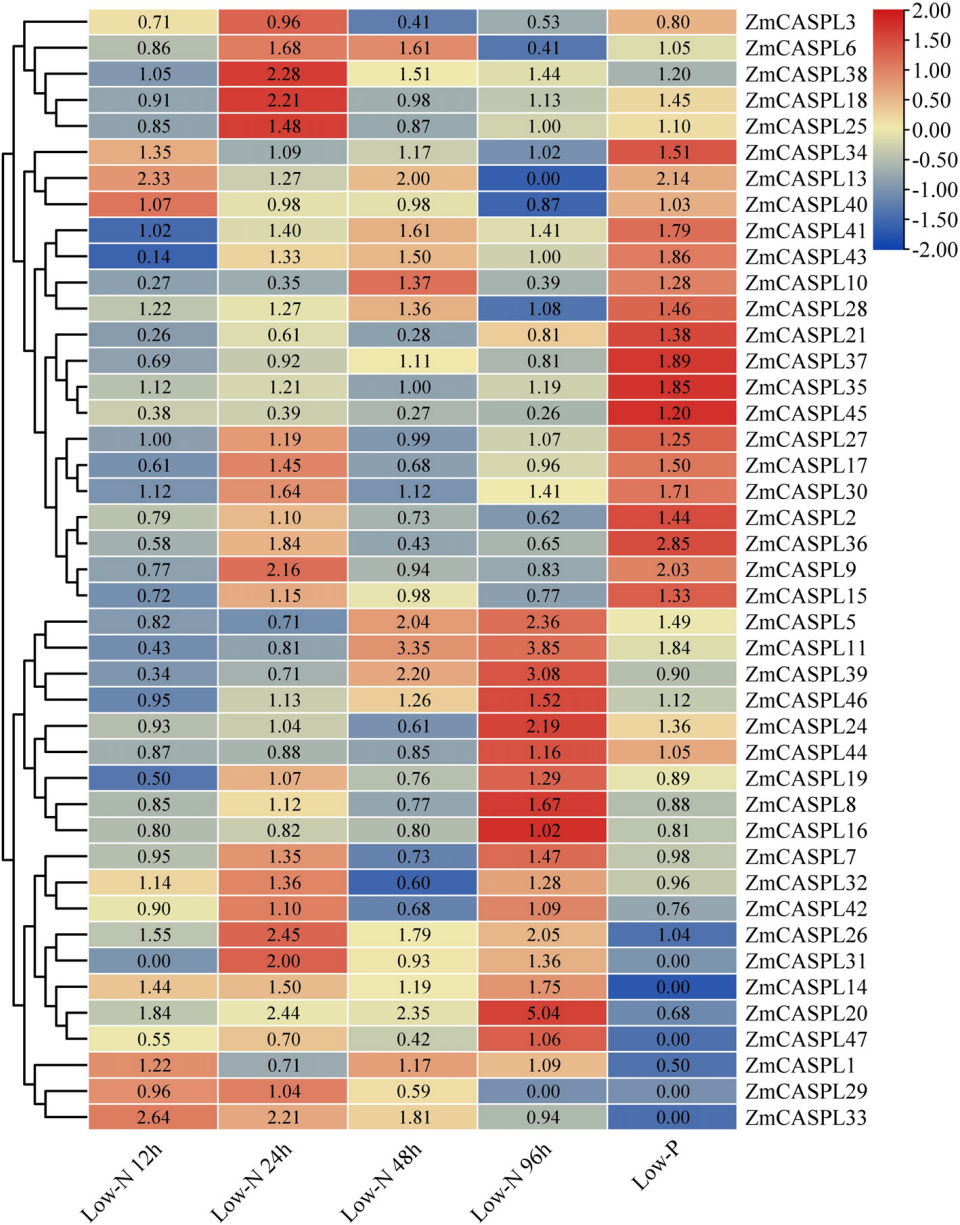
The expression pattern of *ZmCASPL* genes under low nitrogen and phosphorus stresses by RNA-seq. The red and blue boxes indicate high and low expression levels of *ZmCASPL* genes, respectively.

Corn is one of the most significant food crops globally, and it holds the position of being the largest cultivated and highest-yielding grain crop in China. One of the key strategies to boost corn production, reduce production costs, strengthen stress resistance, and promote sustainable agriculture is to explore and harness low-nitrogen and low-phosphorus tolerant genes within corn varieties (Xu et al., 2018; Simons et al., 2014). To mine the potential candidate *ZmCASPL* genes in response to nutrition stress, we analyzed the transcriptome data of maize under low nitrogen and phosphorus stress (**Supplementary Table 6**). As shown in **Figure 9**, our findings revealed that the expression levels of *ZmCASPL5*, *ZmCASPL11*, and *ZmCASPL39* genes were down-regulated at 12 h and 24 h after exposure to low nitrogen, whereas *ZmCASPL20*, *ZmCASPL41*, and *ZmCASPL46* genes were up-regulated at 48 h and 96 h, suggesting their potential roles in the early and later stages of low-nitrogen stress, respectively. Moreover, low-nitrogen treatment can increase the expression of *ZmCASPL14*/*26/30*/*/33*/*34*/*41* genes at all points in time. Notably, the relative expression levels of *ZmCASPL9/15*/*18*/*25*/*26*/*31/38* peaked at 24 h, and subsequently decreased. Interestingly, some *ZmCASPL* genes exhibited the opposite trend after low nitrogen and phosphorus stress. For example, *ZmCASPL7*/*8/16*/*19*/*32*/*39*/*42* were up-regulated under low nitrogen but down-regulated under low phosphorus stress, whereas *ZmCASPL2*/*9/10/15/17/21*/*36/40*/*45* showed the reverse pattern. Additionally, some *ZmCASPL* genes exhibited similar expression patterns after being subjected to low nitrogen and phosphorus treatment. Specifically, the expression patterns of *ZmCASPL26*/*28*/*30*/*34*/*38*/*41* were up-regulated under low nitrogen and phosphorus treatment, while the expression levels of *ZmCASPL3* gene was down-regulated under low nitrogen and phosphorus treatment, respectively. These findings highlight the complex and nuanced responses of *ZmCASPL* genes to different nutrient stresses, underscoring their potential as targets for enhancing maize nutrient use efficiency and stress tolerance.

### The expression patterns of *ZmCASPL* genes in biotic stress

**Figure 10:**
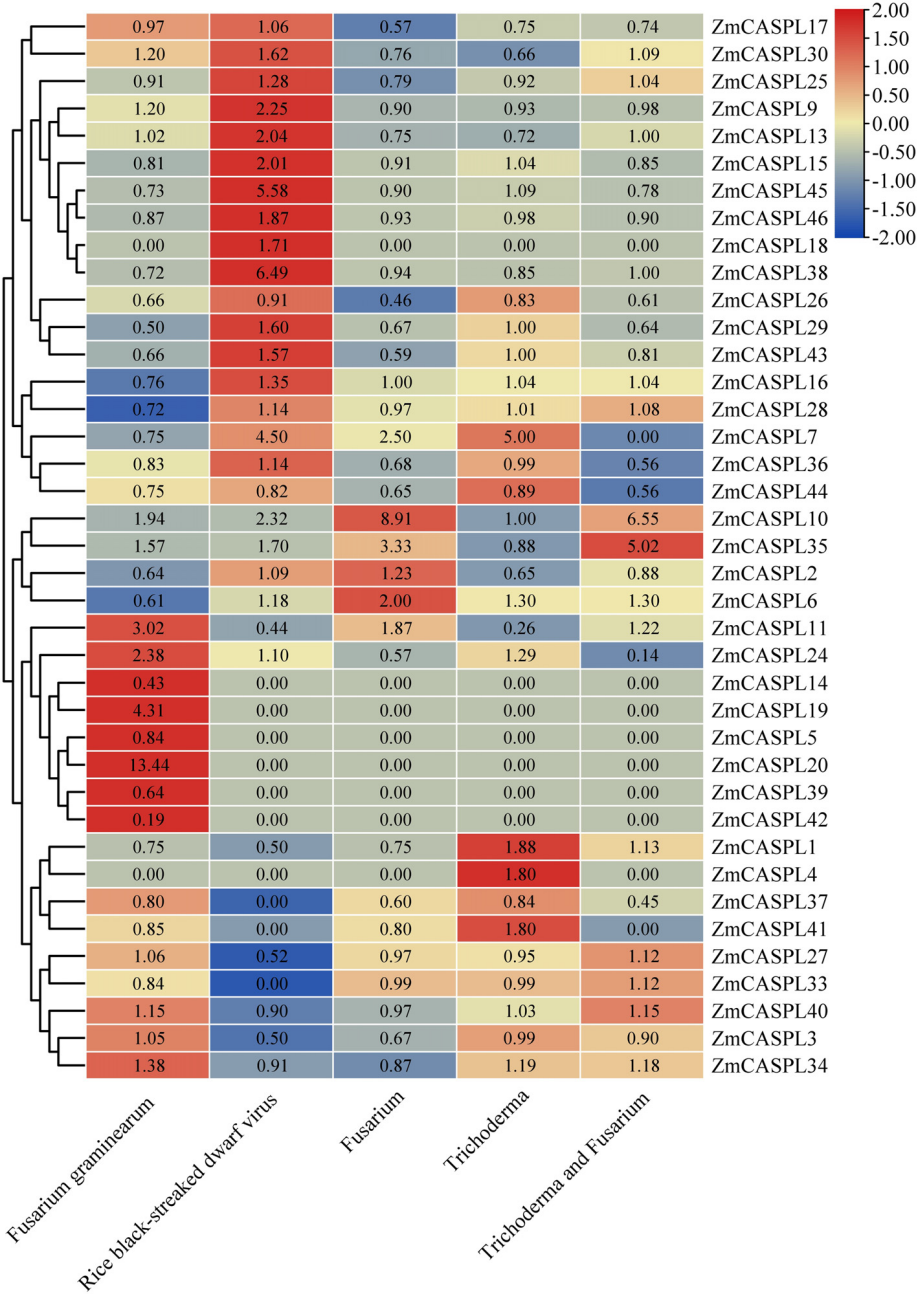
The expression pattern of *ZmCASPL* genes under *Fusarium graminearum*, *Rice black-streaked dwarf virus*, *Fusarium*, *Trichoderma* and *Trichoderma* and *Fusarium* stresses. by RNA-seq. The red and blue boxes indicate high and low expression levels of *ZmCASPL* genes, respectively.

The impact of diseases on corn yield is profound, rendering the exploration and application of disease-resistant genes of paramount significance in enhancing both corn yield and quality (Li et al., 2019; Thompson et al., 2023). To mine the potential candidate *ZmCASPL* genes in response to biotic stress, we analyzed the RNA-seq data of maize under *Fusarium graminearum*, *Rice black-streaked dwarf virus*, *Fusarium*, *Trichoderma* and *Trichoderma* and *Fusarium* stresses (**Supplementary Table 7**). As shown in **Figure 10**, our analysis revealed some *ZmCASPL* genes exhibited the opposite trend after *Fusarium graminearum* and *Rice black-streaked dwarf virus* infection. For example, *ZmCASPL11*, *ZmCASPL14*, *ZmCASPL27*, *ZmCASPL3*, and *ZmCASPL40* exhibited up-regulation under Fusarium graminearum infection but down-regulation under Rice black-streaked dwarf virus infection. Conversely, *ZmCASPL6*/*7*/*15*/*16*/*18*/*28*/*29*/*36*/*38*/*43*/*4*5/*46* displayed the opposite pattern. The expression levels of *ZmCASPL20* were up-regulated (more than 13-old) under *Fusarium graminearum* infection. These results suggest that the function of *ZmCASPL* genes is different after infection by *Fusarium graminearum* and *Rice black-streaked dwarf virus*. Interestingly, we found that some *ZmCASPL* genes under *Fusarium*, *Trichoderma* and *Trichoderma* and *Fusarium* stresses, the expression level first was up-regulated, then decreased, and then up-regulated, such as *ZmCASPL10*, *ZmCASPL11* and *ZmCASPL35*. On the contrary, some *ZmCASPL* genes under *Fusarium*, *Trichoderma* and *Trichoderma* and *Fusarium* stresses, the expression level of *ZmCASPL1*, *ZmCASPL15 ZmCASPL24*, and *ZmCASPL41* first was down-regulated, then increased, and then down-regulated. These findings underscore the dynamic and nuanced responses of *ZmCASPL* genes to different microbial challenges and their potential importance in mediating plant defense mechanisms.

### Expression pattern analysis of *ZmCASPL* genes under PEG and salt treatments by RT-qPCR

**Figure 11:**
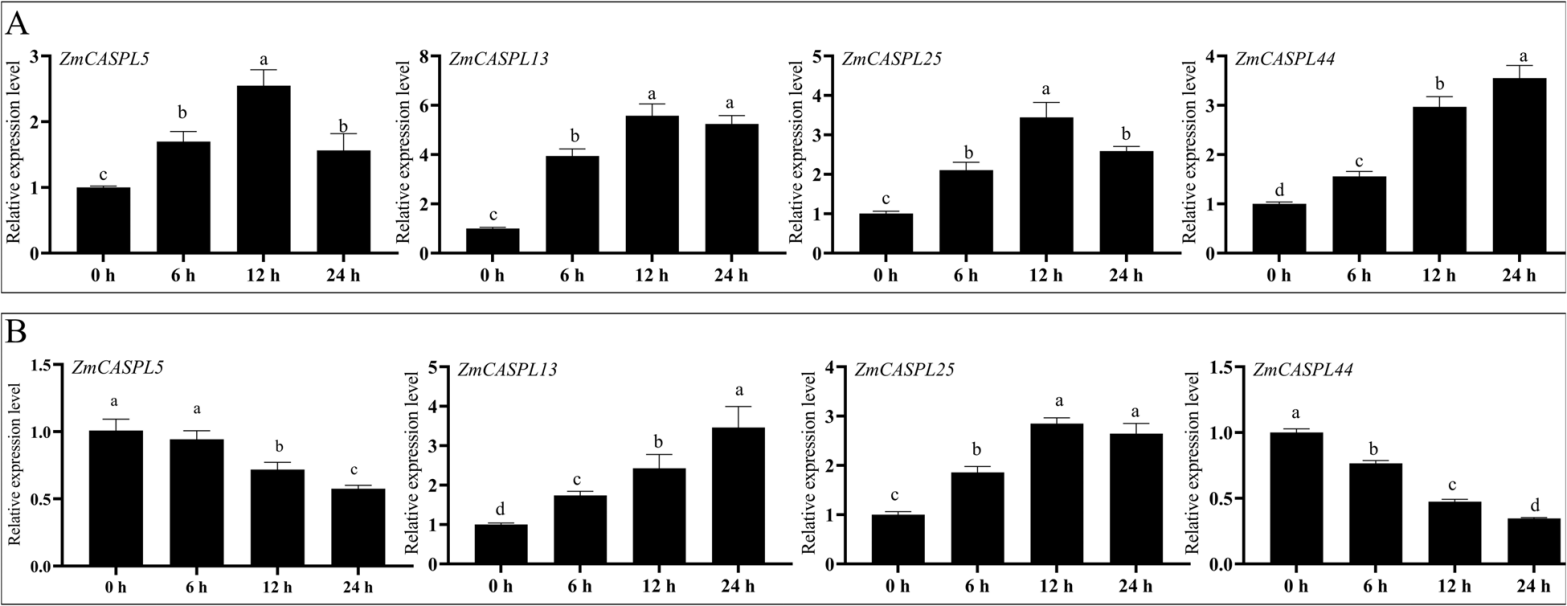
The relative expression level of *ZmCASPL* genes under PEG6000 and NaCl treatment by RT-qPCR. **(A)** The relative expression level of *ZmCASPL5*, *ZmCASPL13*, *ZmCASPL25* and *ZmCASPL44* genes under 30% PEG6000 treatment. **(B)** The relative expression level of *ZmCASPL5*, *ZmCASPL13*, *ZmCASPL25* and *ZmCASPL44* genes under 150 mM NaCl treatment. The expression levels were calculated using 2^-△△Ct^ methods. Means ± SDs (n = 3). Different lowercase letters indicate significant differences among means (P<0.05), as calculated with Fisher,s protected LSD test.

To further explore the potential function of *ZmCASPL* genes, we selected four *ZmCASPL* genes that exhibited responsiveness to both drought and salt stress based on RNA-seq data. As shown in **Figure 11**, these genes have different expressions after 30% PEG6000 (to mimic drought stress) and 150 mM NaCl (to simulate salt stress). Intriguingly, the relative expression levels of *ZmCASPL5*, *ZmCASPL13*, *ZmCASPL25* and *ZmCASPL44* were significantly up-regulated in 30% PEG6000 treatment, with peak expressions observed at 12 h, 12 h, 12 h and 24 h, respectively (**Figure 11**). Conversely, under 150 mM NaCl treatment, the expression levels of *ZmCASPL13* and *ZmCASPL25* were up-regulated, whereas the expression level of *ZmCASPL5* and *ZmCASPL44* displayed a down-regulation. Moreover, after PEG and NaCl treatments, we found that *ZmCASPL13* and *ZmCASPL44* genes had an opposing expression pattern (**Figure 11**). Specifically, their expression levels were up-regulated following 30% PEG6000 treatment but down-regulated upon salt stress treatment. These results suggest the complexity and specificity of the stress-responsive pathways of *ZmCASPL* genes.

### Gene co-expression analysis

Gene regulatory network is a complex system involving the interaction of genes, proteins, small molecule signals and other molecules. Through co-expression network analysis, we can deeply understand the regulatory relationships among these molecules, and provide important clues for understanding the regulatory mechanisms of biological systems. In this study, we used the MaizeNetome database (Feng et al., 2023) to construct a co-expression network centered on the ZmCASPL5, ZmCASPL13, ZmCASPL25 and ZmCASPL44. As shown in **Supplementary Figure 1**, we obtained a total of eight co-expression networks. Among them, the network centered on ZmCASPL13 is the largest (209 genes and 24 Lnc RNA). In contrast, the network centered on ZmCASPL44 is the smallest (1 genes). As shown in **Supplementary Table 8**, the co-expressed gene network centered on ZmCASPL5 is significantly enriched in heavy metal transport process, lignin polymerization process, ATP-binding cassette subfamily G transporters and ethylene signal transduction pathway. Moreover, the network centered around ZmCASP13 showed significantly enriched in histone modification, ATP-binding cassette sub-family B/C, photosynthesis, response to oxidative stress, cell wall organization process, oxidative phosphorylation process and response to salt and drought stress. On the other hand, ZmCASPL44 showed significant enrichment in response to heat stress; phosphoinositide binding, calcium signaling pathway, oxidative phosphorylation process, carbohydrate metabolic process and cell wall biogenesis.

## Discussion

The CASPs protein family performs a multitude of crucial roles in plants, encompassing the formation of the Casparian strip, the absorption of mineral elements, the regulation of growth and development, as well as the response to environmental stresses (Roppolo et al., 2011; Wang et al., 2019; Yang et al., 2015). Collectively, these functions serve to safeguard the normal growth and development of plants, thereby enhancing their adaptability and resilience to various environmental conditions. However, the function of *ZmCASPL* gene in maize has not been fully studied. In this study, a total of 47 ZmCASPL members were confirmed in maize. Previous studies have shown that the *CASPL* gene is widely present in plants. Such as, 156 members were confirmed in *Pogostemon cablin* (Su et al., 2023), 39 in *Arabidopsis* (Roppolo et al., 2014), 48 in *G. arboretum* (Wang et al., 2020), 91 in *G. barbadense* (Wang et al., 2020), 94 in *G. hirsutum* (Wang et al., 2020) and 54 in *G. raimondii* (Wang et al., 2020), respectively. The number of *CASPL* was more than that of *Arabidopsis* and less than 56 *Pogostemon cablin*, 48 in *G. arboreum*, 91 in *G. barbadense*, and 94 in *G. hirsutum*. The likely reason that *G. arboreum*, *G. barbadense*, *G. hirsutum*, *G. raimondii* and *Pogostemon cablin* have more *CASPL* genes is that it has a more complex genome than *Arabidopsis* and maize (Zhang et al., 2015).

The TD and WGD events are two crucial mechanisms in genomic evolution, each playing distinct roles in the process of biological evolution (Freeling, 2009; Panchy et al., 2016). TD serves as a pivotal source of genetic diversity for organisms, enabling them to adapt to diverse environmental pressures (Holland, 2013). Through the processes of gene duplication, followed by mutations, selections, and other evolutionary mechanisms, organisms can evolve novel gene functions and traits. WGD, also known as polyploidy, refers to the duplication of all sequences within a genome (Freeling, 2009). This mode of replication holds paramount significance in biological evolution, as it furnishes species with an abundant pool of genetic material, thereby catalyzing rapid genome reorganization and advancement. Previous studies have speculated that maize undergoes at least three genome-wide replication events; and these repeated events occurred about 110 million years ago, 50 million years ago and 12 million years ago (Lyons and Freeling, 2008; Tang et al., 2008; Lee et al., 2013). Through collinearity analysis, 11 TD and 18 WGD *ZmCASPL* genes were identified in maize. These results suggest that TD and WGD could have contributed to the expansion of the *ZmCASP* gene family in maize, but WGD may have played a major role. Interestingly, we found that there were more WGD genes in Group V and Group VI, suggesting that the expansion of *ZmCASPL* gene family mainly occurred in Group V and Group VI.

The intricate relationship that exists between gene structure and gene function underscores the importance of delving deeply into the former (Shaul, 2017). By conducting thorough investigations into gene structure, we can gain a more profound understanding of gene function and its regulatory mechanisms. Based on conserved motifs and gene structure analysis, we found that the same Group of ZmCASPL contains similar gene structure and conserved motifs, implying that these *ZmCASPL* genes may have similar functions and regulatory mechanisms. On the other hand, we found the same subfamily has a similar tissue expression profile of *ZmCASPL* genes in maize. A series of studies have revealed that CASPL family genes are widely expressed in various tissues, with similar expression patterns in the same subfamily observed in cotton Arabidopsis. The results of the tissue expression pattern showed that among the 47 *ZmCASPL* genes, 43 were expressed in the roots and 14 were highly expressed in the roots, *ZmCASPL7/8/18/19/21/24/32/47* are specifically highly expressed in the root, and *ZmCASPL21* and *ZmCASPL47* are only highly expressed in the root. These results prompted us to hypothesize that *ZmCASPL21* and *ZmCASPL47* may play an important role in Casparian strip development in maize. Previous studies have shown the presence of CS structures in the stems and leaves of ferns (Lersten, 1997). Recent studies have for the first time identified a novel apoplastic barrier cell wall structure characterized by lignin-rich neck strips that specifically regulate the formation of cucumber peel wax powder in non-root cells (Hao et al., 2024). Based on these findings, our study found that some *ZmCASPL* genes are highly expressed in leaves. Given the functional diversity of *ZmCASPL* genes, means that they may be involved in the development of leaf CS, thereby affecting leaf structure, function and possibly plant adaptability. Therefore, we emphasize the importance of follow-up studies to test this hypothesis and elucidate the specific role of *ZmCASPL* genes in leaf CS development.

Remarkably, our investigation uncovered a significant abundance of light-responsive elements within the promoter regions of *ZmCASPL* genes. These findings suggest that the *ZmCASPL* gene not only serves as a pivotal player in the developmental processes of the CS and stress adaptation, but also holds a crucial function in the intricate process of photogenesis, a mechanism that warrants further in-depth exploration.

In the field cultivation environment, the yield and quality of corn are confronted with multiple environmental challenges (Simons et al., 2014; Xu et al., 2018; Li et al., 2019; Sun et al., 2023; Thompson et al., 2023). Drought conditions constrain the effective water supply, thereby impeding the normal growth and development of the plants (Muthuvel et al., 2023). On the other hand, high salt, hinders the absorption of water and nutrients by corn roots, further diminishing its growth potential (Zheng et al., 2024). Extreme weather conditions, both cold and heat, exert pressure on the physiological functions of corn, leading to reduced yields (Zheng et al., 2024; Jiang et al., 2022). Moreover, low nitrogen and phosphorus levels in the soil directly limit corn’s nutrient uptake, subsequently affecting its growth rate and ultimate yield (Xu et al., 2018). Of even greater concern, the invasion of pathogenic microorganisms can trigger various diseases, posing a direct threat to both the yield and quality of corn (Li et al., 2019). Previous studies have shown that *CASPL* gene plays an important role in abiotic and biological stress processes (Roppolo et al., 2011; Yang et al., 2015; Wang et al., 2019). In this study, by analyzing the transcriptome data of maize treated with drought, salt, heat, cold, low nitrogen, and low phosphorus stresses and pathogen infection, we found that *CASPL* gene had different expression patterns under different stress treatments. Among them, *ZmCASPL5/30/44/46* were down-regulated and up-regulated under salt and drought stress, respectively. In addition, the CS has been proved to play an important role in plant resistance to drought, salt, cold, heavy metals and other stresses. Wang et al. revealed the important mechanism of maize dirigent protein ZmSTL1/ZmESBL in promoting salt stress adaptation by regulating the development of endodermal CS (Wang et al., 2022). We hypothesize that these genes may be involved in the development of the CS, and thus increase salt tolerance and drought stress tolerance by altering the root diffusion barrier. This interesting phenomenon deserves further investigation. Moreover, *ZmCASPL7/8/16/19/32/3/42* were up-regulated and down-regulated under low nitrogen and phosphorus stress, respectively. On the contrary, *ZmCASPL2/9/10/15/17/21/36/40/37/45* were down-regulated and up-regulated under low nitrogen and phosphorus stress, respectively. These *ZmCASPL* genes may be involved in the processes of suberization and lignification in maize, and affects nitrogen and phosphorus balance and plant development and growth. It’s worth noting that *ZmCASPL11/14/27/3/40* were up-regulated and down-regulated under *Fusarium graminearum* and *Rice black-streaked dwarf virus* infection, respectively. However, *ZmCASPL6/7/15/18/16/28/29/36/38/43/46* were down-regulated and up-regulated under low nitrogen and phosphorus stress, respectively. The expression levels of *ZmCASPL20* were up-regulated (more than 13-old) under *Fusarium graminearum* infection. Suggesting *ZmCASPL* genes potential value in maize enhancing stress resilience breeding, but the specific biological functions still require further exploration.

## Methods

### Identification and phylogenetic analysis of *ZmCASPL*

The CDS sequences, protein sequences, genome sequence and genome annotation (GFF) file of *Z. mays* was obtained from the online database phytozome v13 (https://Phytozome-next.jgi.doe.gov) (Goodstein et al., 2012). Firstly, The HMM (Hidden Markov Model) (Potter et al., 2018) of the DUF588 domain (PF04535) was downloaded from the online PFAM database (Mistry et al., 2021) (http://pfam.sanger.ac.uk/ ), and use HMMER software to search for ZmCASPL genes in the *Z. mays* protein database. Secondly, according to the BLASTP method, we searched ZmCASPL protein sequences using AtCASPL protein sequences. Then, ZmCASPL protein sequences were submitted to the online website NCBI-CDD (https://www.ncbi.nlm.nih.gov/Structure/bwrpsb/bwrpsb.cgi) to identify the conserved DUF588 and MARVEL domain. The chromosome distribution of *ZmCASPL* genes was obtained from the GFF file. The phylogenetic analysis was performed of AtCASPL and ZmCASPL protein sequences by the neighbor-joining (N-J) method (Kumar et al., 2016). Lastly, the picture was embellished through the online website iTOL (https://itol.embl.de/) (Letunic and Bork, 2021).

### Gene structure and conserved motifs analysis of *ZmCASPL*

The gene structure, conserved motifs and conserved domain of ZmCASPL were obtained from the GFF file, online website MEME (Bailey et al., 2009) and online website NCBI-CDD, respectively. The picture of the phylogenetic tree, conserved motifs, conserved domain and gene structure was visualized through TBtools software (Chen et al., 2023).

### Duplication events and Ka/Ks analysis of *ZmCASPL*

The WGD and TD events were analyzed by MCScan (Wang et al., 2012). The TBtools were used to analyze ka, ks and Ka/Ks ratios, and homologous gene pairs (Chen et al., 2023).

### Cis-acting element analysis of *ZmCASPL*

The cis-elements of ZmCASPL genes promoter regions were analyzed by Plant Care (http://bioinformatics.psb.ugent.be/webtools/plantcare/html/) (Lescot et al., 2002).

### Expression patterns of *ZmCASPL* with RNA-seq

The transcriptome data of maize in different tissues (root, endosperm, ear, embryo, leaf, shoot, leaf base, leaf tip and anther) were downloaded from the Plant Public RNA-seq Database (PPRD, http://ipf.sustech.edu.cn/pub/plantrna/) (PRJEB35943) (Yu et al., 2022b). The transcriptome data of maize under drought (PRJNA378714), salt, heat and cold (PRJNA244661) treatment were downloaded from the PPRD database. The transcriptome data of maize under low nitrogen (PRJNA304223) and phosphorus (PRJNA269060) treatment were downloaded from the PPRD database. The transcriptome data of maize under *Fusarium graminearum* (PRJNA357594), *Rice black-streaked dwarf virus* (PRJNA438075), *Fusarium*, *Trichoderma* and *Trichoderma* and *Fusarium* (PRJNA362306) stresses were downloaded from the PPRD database. The gene expression fold was calculated using FPKM.

The maize breed was B73; these tissues were collected at different development stage, including embryo (16 days after pollination), endosperm (16 days after pollination), anther (first flower open stage), ear (eighteen leaves visible stage), leaf tip (eleven leaves visible stage), leaf (eleven leaves visible stage), leaf base (eleven leaves visible stage), shoot (seedling) and root (seedling).

For the gene expression patterns of different abiotic stresses (cold, salt, drought and flood), After 16 days of germination, the seedlings were subjected to a water deficient, salt, cold and heat treatments. then samples were obtained after the drought treatment. For the drought treatment, the seedlings samples were obtained for the drought treatment 6 days. For salt stress, the seedlings samples were obtained for the salt treatment 2 h. For heat and cold stress treatments, seedlings were incubated at 42°C and 4°C for 2 h, respectively, then the seedlings samples were collected.

Under low nitrogen, the seedlings were subjected to a low nitrogen treatment, then samples were obtained after the low nitrogen treatment 0 h, 12 h, 24 h, 48 h and 96 h. For low phosphorus treatment, after 10 days of germination, plants were fertilized with Hoagland solution containing the 1000 μM (+P) or 10 μM (–P) concentration of KH_2_PO_4_. The roots were collected of three-leaf seedling. For *Fusarium graminearum* inoculation, 6–8 sib-crossed primary ears from each inbred were inoculated with 1 ml of fungal inoculum on the same day prior to 10 am and a similar number of ears were injected with Bilay’s media as a mock treatment, and samples were obtained for the *Fusarium graminearum* inoculation 11 days. For *Rice black-streaked dwarf virus* treatment, the leaves of one-month-old maize seedlings with rough dwarf disease symptoms were collected.

### Plant materials, growth conditions and PEG and NaCl treatments

The maize Seedlings were cultivated in a greenhouse under the conditions of 28/22°C during a 14/10 h light/dark cycle (200 mmol m^−2^ s^−1^) and 27°C. The seeds after three days of germination, the seeds were transferred to black boxes containing Hoagland nutrient solution (Zhu et al., 2022), and the Hoagland nutrient solution were replaced every three days.

For the 30% PEG6000 and 150 mM NaCl treatments, the maize seedlings were cultured with Hoagland nutrient solution for three-leaf stage, then transferred into normal solution, 30% PEG6000 and 150 mM NaCl, respectively. Samples were obtained collected at 0 h, 6 h, 12 h and 24 h after initiation of the stress treatment.

### Total RNA extraction and RT-qPCR analysis

In this study, we u*s*ed primer 3.0 (https://primer3.ut.ee/) online website to design *ZmCASPL* gene*-*specific RT-qPCR primer (**Supplementary Table 9**). The maize root total RNA were extracted by E.Z.N.A.^®^ Plant RNA Kit (omega, Norcross,Georgia, America). The cDNA was reverse transcription synthesis by PrimeScript™ RT reagent Kit (Perfect Real Time) (TaKaRa, Nojihigashi, Japan). The RT-qPCR reaction system and RT-qPCR amplification program (Xue et al., 2024).

## Conclusions

In this study, 47 *ZmCASPL* genes were identified in maize genome. The phylogenetic tree revealed that AtCASPL and ZmCASPL are divided into six Groups. Moreover, we found that the same Group of ZmCASPL contains similar gene structure and conserved motifs. WGD is the main driving force of CASPL gene family expansion in maize. The tissue expression pattern and Cis-elements analysis showed *ZmCASPL21* and *ZmCASPL47* may play an important role in CS development. Furthermore, we found *ZmCASPL* may play a crucial role in the maize response to abiotic stresses, biotic stresses and nutritional deficiency defect. Under PEG6000 and NaCl treatment, *ZmCASPL13/25* showed an opposite expression pattern after, while *ZmCASPL5/44* showed a similar expression pattern. This study laid a foundation for further study on the function of *ZmCASPL* genes in maize, and even other plants.

## Data Availability

The data presented in the study are deposited in the BioProject repository, accession number: PRJEB35943, PRJNA378714, PRJNA244661, PRJNA304223, PRJNA269060, PRJNA357594, PRJNA438075 and PRJNA362306.

## Supplementary material

Supplementary Table 8 Co-expression network analysis of *ZmCASPL* genes.

Supplementary Table 9 The Primer sequences of *ZmCASPL* genes used for RT-qPCR.
