# Supplementary material for: Genome-wide identification and expression analysis of CASPL gene family in Zea mays (L.)
Source: Front Plant Sci. 2024 Oct 28;15:1477383. doi: 10.3389/fpls.2024.1477383 (PMC11550983; doi:10.3389/fpls.2024.1477383)
Supplement: Supplementary Table 8 — Co-expression network analysis of ZmCASPL genes. [file Table8.docx]

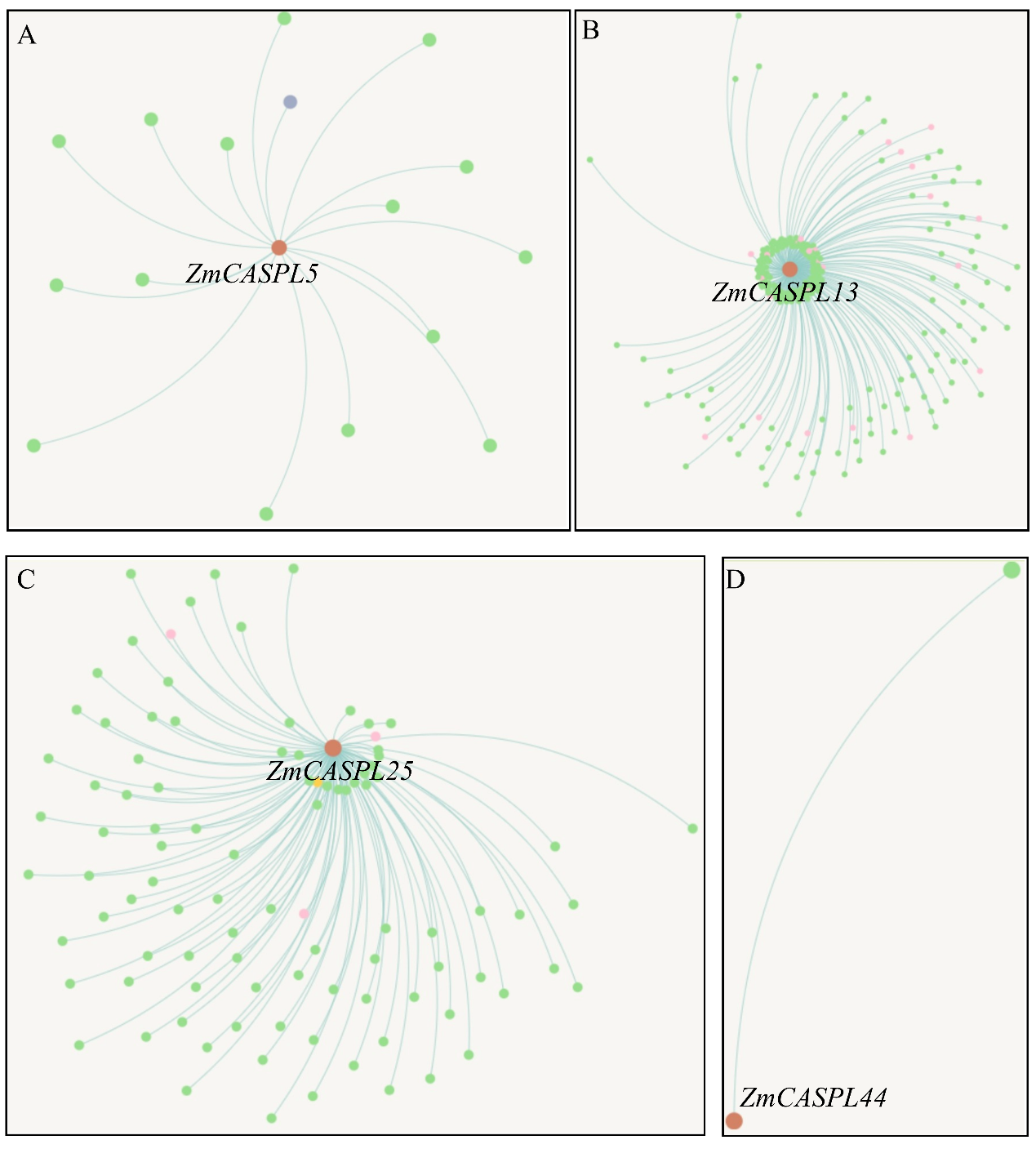


Supplemental Figure 1. Co-expression network of ZmCASPL5 (A), ZmCASPL13 (B), ZmCASPL25 (C) and ZmCASPL44 (D). Green dots represent genes, and lines indicate that they have co-expression relationship.
